# Supplementary material for: PAM50 Intrinsic Subtype Profiles in Primary and Metastatic Breast Cancer Show a Significant Shift toward More Aggressive Subtypes with Prognostic Implications
Source: Cancers (Basel). 2021 Mar 30;13(7):1592. doi: 10.3390/cancers13071592 (PMC8037951; doi:10.3390/cancers13071592)
Supplement: Supplementary file 1 [file cancers-13-01592-s001.pdf]

cancers

MDPI

Supplementary Materials: PAM50 Intrinsic Subtype Profiles in Primary and Metastatic Breast Cancer Show a Significant Shift toward More Aggressive Subtypes with Prognostic Implications

Charlotte Levin Tykjær Jørgensen, Anna-Maria Larsson, Carina Forsare, Kristina Aaltonen, Sara Jansson, Rachel Bradshaw, Pär-Ola Bendahl and Lisa Rydén

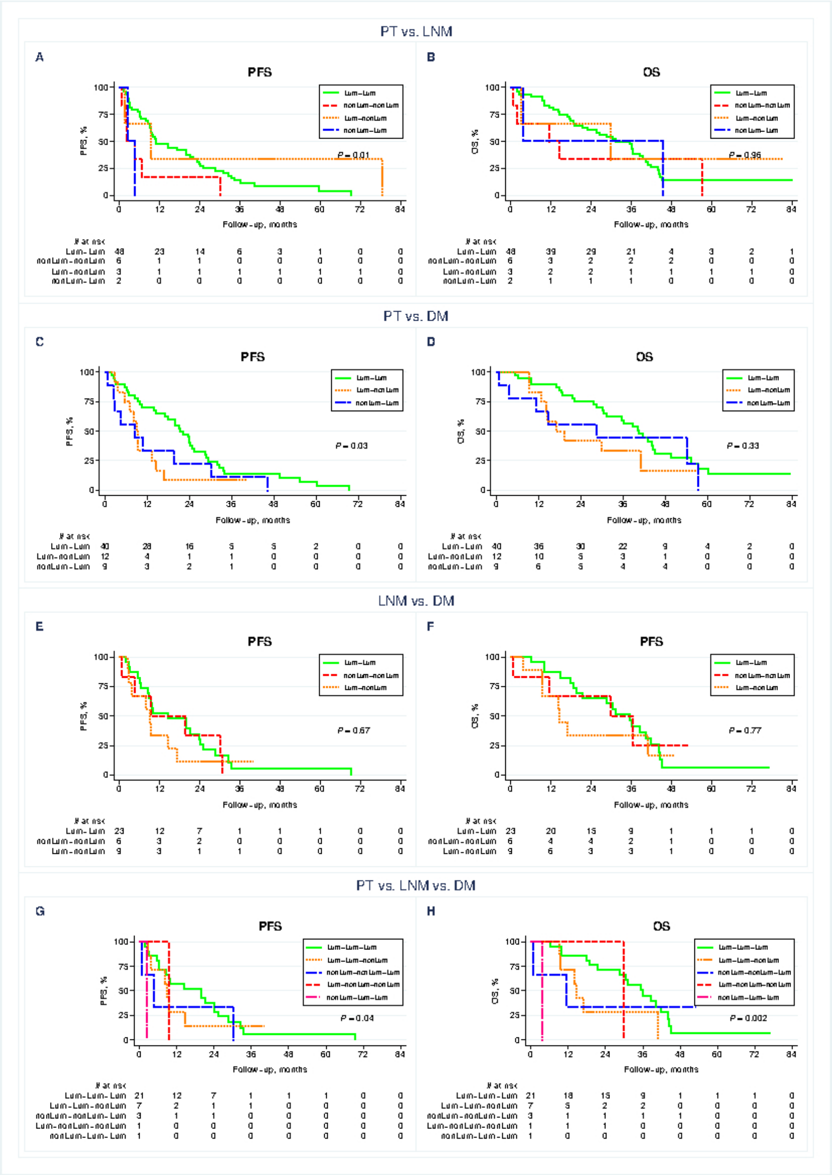

Figure S1: Kaplan–Meier survival curves for progression-free survival and overall survival according to luminal versus non-luminal subtype subgroups in the primary tumor, lymph node metastasis, and distant metastasis. *p*-values were calculated with the log-rank test

Table S1. Patient demographics, disease characteristics, and prior therapy given for the entire CTC–MBC cohort and for patients included/excluded in the present study.

Characteristics	Entire CTC-MBC Cohort *n* (%)	Included in PAM50 Cohort *n* (%)	PAM50 Subtype Available *n* (%)
	156 (100)	140 (90)	123 (79)
Age at MBC diagnosis (years)			
<65	75 (48)	70 (50)	63 (51)
≥65	81 (52)	70 (50)	60 (49)
ECOG (baseline)			
0	91 (60)	82 (60)	70 (58)
1	37 (25)	35 (25)	31 (26)
2	22 (15)	20 (15)	19 (16)
Missing	6	3	3
Primary tumor size			
T1	57 (39)	47 (36)	38 (33)
T2	51 (35)	48 (36)	43 (37)
T3	20 (13)	20 (15)	19 (16)
T4	19 (13)	17 (13)	17 (14)
Missing	9	8	6
Nodal status[a]			
Negative	44 (32)	36 (30)	31 (29)
Positive	92 (68)	87 (70)	77 (71)
Missing	20	17	15
NHG (PT)			
I	13 (11)	11 (10)	8 (8)
II	65 (52)	59 (53)	50 (51)
III	46 (37)	42 (37)	40 (41)
Missing	32	28	25
ER (PT)			
Negative	24 (16)	22 (17)	20 (17)
Positive	123 (84)	110 (83)	99 (83)
Missing	9	8	4
PR (PT)			
Negative	45 (31)	42 (32)	37 (32)
Positive	100 (69)	88 (68)	80 (68)
Missing	11	10	6
HER2 (PT)			
Negative	109 (86)	96 (85)	89 (86)
Positive	18 (14)	17 (15)	14 (14)
Missing	29	27	20
BC subtype (PT)			
ER+ HER2-	92 (73)	81 (72)	75 (73)
HER2+	17 (13.5)	16 (14)	13 (13)
ER- HER2-	17 (13.5)	15 (14)	14 (14)
Missing	30	28	21
BC subtype (REC/distant met)			
ER+ HER2-	80 (70)	73 (70)	64 (72)
HER2+	16 (14)	16 (15)	12 (13)
ER- HER2-	18 (16)	16 (15)	13 (15)
Missing	42	35	34
MFI (years)			
0	31 (20)	29 (21)	26 (21)
>0-3	28 (18)	25 (18)	23 (19)
>3	97 (62)	86 (61)	74 (60)

Number of metastatic sites			
1–2	109 (70)	96 (69)	82 (67)
≥3	47 (30)	44 (31)	41 (33)
Metastatic site			
Non-visceral	65 (42)	58 (41)	51 (42)
Visceral	91 (58)	82 (59)	72 (58)
Number of CTCs (BL)			
<5	73 (48)	66 (48)	57 (46)
≥5	79 (52)	73 (52)	66 (54)
Missing	4	1	0
Prior Therapy[b]			
Neoadjuvant chemo	27 (17)	26 (19)	25 (20)
Adjuvant chemo	71 (46)	66 (47)	57 (46)
Adjuvant endocrine	96 (62)	85 (61)	75 (61)
Adjuvant HER2-targeted	5 (3)	4 (3)	2 (2)

Abbreviations: BL, baseline; BC, breast cancer; CTC, circulating tumor cell; ECOG, Eastern Cooperative Oncology Group; ER, estrogen receptor; HER2, human epidermal growth factor receptor 2; MBC, metastatic breast cancer; MFI, metastasis-free interval; *n*, number; NHG, Nottingham Histological Grade; PR, progesterone receptor; PT, primary tumor; REC, recurrence; [a]At primary diagnosis; [b]Some patients were given more than one type of therapy, hence the percentages in parentheses sum up to more than 100.

Table S2. Patient and tumor characteristics for the lymph node metastases in the PAM50 cohort with subtypes available and subdivided into the different breast cancer intrinsic subtypes.

**Characteristics**	**Subtype Available *n* (%)**	**Lum A *n* (%)**	**Lum B *n* (%)**	**HER2-E *n* (%)**	**Basal-Like *n* (%)**	***P*[a]**
	68	18	37	9	4	
Age at MBC diagnosis (years)						0.96
<65	27 (40)	7 (39)	14 (38)	4 (44)	2 (50)	
≥65	41 (60)	11 (61)	23 (62)	5 (56)	2 (50)	
ECOG (BL)						0.16
0	43 (65)	15 (88)	22 (61)	4 (44)	2 (50)	
1	16 (24)	1 (6)	10 (28)	4 (44)	1 (25)	
2	7 (11)	1 (6)	4 (11)	1 (12)	1 (25)	
Missing	2	1	1	0	0	
Size (PT)						0.16
T1	16 (25)	4 (24)	9 (25)	2 (22)	1 (33)	
T2	28 (43)	3 (17)	19 (53)	5 (56)	1 (33)	
T3	13 (20)	6 (35)	6 (17)	1 (11)	0	
T4	8 (12)	4 (24)	2 (5)	1 (11)	1 (33)	
Missing	3	1	1	0	1	
Nodal status[b]						
Negative	0					
Positive	65 (100)	18 (100)	35 (100)	9 (100)	3 (100)	
Missing	3	0	2	0	1	
NHG (PT)						0.45
I	5 (8)	2 (13)	3 (9)	0	0	
II	37 (61)	11 (74)	20 (59)	5 (56)	1 (33)	
III	19 (31)	2 (13)	11 (32)	4 (44)	2 (67)	
Missing	7	3	3	0	1	
ER (PT)						0.003
Negative	7 (11)	0	2 (6)	3 (33)	2 (67)	
Positive	57 (89)	16 (100)	34 (94)	6 (67)	1 (33)	
Missing	4	2	1	0	1	

PR (PT)						0.08
Negative	19 (31)	2 (13)	10 (29)	5 (56)	2 (67)	
Positive	43 (69)	13 (87)	25 (71)	4 (44)	1 (33)	
Missing	6	3	2	0	1	
HER2 (PT)						0.02
Negative	44 (85)	11 (100)	27 (90)	5 (56)	1 (50)	
Positive	8 (15)	0	3 (10)	4 (44)	1 (50)	
Missing	16	7	7	0	2	
BC subtype PT						<0.001
ER+ HER2-	39 (75)	11 (100)	26 (87)	2 (22)	0	
HER2+	8 (15)	0	3 (10)	4 (45)	1 (50)	
ER- HER2-	5 (10)	0	1 (3)	3 (33)	1 (50)	
Missing	16	7	7	0	2	
MFI (years)						0.03
0	6 (9)	1 (5)	4 (11)	0	1 (25)	
>0-3	14 (20)	3 (17)	4 (11)	5 (56)	2 (50)	
>3	48 (71)	14 (78)	29 (78)	4 (44)	1 (25)	
Number of metastatic sites						0.71
1–2	46 (68)	11 (61)	27 (73)	6 (67)	2 (50)	
≥3	22 (32)	7 (39)	10 (27)	3 (33)	2 (50)	
Metastatic site						0.67
Non-visceral	27 (40)	6 (33)	14 (38)	5 (56)	2 (50)	
Visceral	41 (60)	12 (67)	23 (62)	4 (44)	2 (50)	
Number of CTCs (BL)						0.41
<5	28 (42)	10 (56)	15 (41)	2 (22)	1 (33)	
≥5	39 (58)	8 (44)	22 (59)	7 (78)	2 (67)	
Missing	1	0	0	0	1	

Abbreviations: BL, baseline; CTCs, circulating tumor cell; ECOG, Eastern Cooperative Oncology Group; ER, estrogen receptor; HER2, human epidermal growth factor receptor 2; HER2-E, human epidermal growth factor receptor 2 enriched; MBC, metastatic breast cancer; MFI, metastasis-free interval; NHG, Nottingham Histological Grade; *n*, number; PT, primary tumor PR, progesterone receptor. [a]*P*-value from Fisher's exact test; [b]At primary diagnosis

Table S3. Patient and tumor characteristics for the distant metastases in the PAM50 cohort with subtype available and subdivided into the different breast cancer intrinsic subtypes.

**Characteristics**	**Subtype Available *n* (%)**	**Lum A *n* (%)**	**Lum B *n* (%)**	**HER2-E *n* (%)**	**Basal-Like *n* (%)**	***P*[a]**
	74	7	41	19	7	
Age at MBC diagnosis (years)						0.41
<65	34 (46)	3 (43)	16 (39)	10 (53)	5 (71)	
≥65	40 (54)	4 (57)	25 (61)	9 (47)	2 (29)	
ECOG (BL)						0.41
0	38 (53)	2 (29)	18 (46)	13 (68)	5 (71)	
1	21 (29)	4 (57)	12 (31)	4 (21)	1 (14.5)	
2	13 (18)	1 (14)	9 (23)	2 (11)	1 (14.5)	
Missing	2	0	2	0	0	
Size (PT)						0.31
T1	29 (41)	1 (14)	17 (42)	8 (47)	3 (43)	
T2	30 (42)	3 (43)	18 (45)	7 (41)	2 (29)	
T3	8 (11)	1 (14)	4 (10)	2 (12)	1 (14)	

T4	4 (6)	2 (29)	1 (3)	0	1 (14)	
Missing	3	0	1	2	0	
Nodal status[b]						0.80
Negative	21 (30)	1 (17)	12 (30)	5 (31)	3 (43)	
Positive	48 (70)	5 (83)	28 (70)	11 (69)	4 (57)	
Missing	5	1	1	3	0	
NHG (PT)						1.00
I	6 (9)	0	4 (11)	2 (12)	0	
II	41 (64)	4 (80)	23 (62)	10 (59)	4 (80)	
III	17 (27)	1 (20)	10 (27)	5 (29)	1 (20)	
Missing	10	2	4	2	2	
ER (PT)						<0.001
Negative	11 (16)	0	0	5 (26)	6 (86)	
Positive	57 (84)	7 (100)	35 (100)	14 (74)	1 (14)	
Missing	6	0	6	0	0	
PR (PT)						0.009
Negative	20 (30)	0	7 (21)	8 (42)	5 (71)	
Positive	47 (70)	7 (100)	27 (79)	11 (58)	2 (29)	
Missing	7	0	7	0	0	
HER2 (PT)						0.05
Negative	53 (89)	7 (100)	28 (97)	13 (72)	5 (83)	
Positive	7 (11)	0	1 (3)	5 (28)	1 (17)	
Missing	14	0	12	1	1	
BC subtype PT						<0.001
ER+ HER2-	45 (76)	7 (100)	28 (100)	9 (50)	1 (17)	
HER2+	6 (10)	0	0	5 (28)	1 (17)	
ER- HER2-	8 (14)	0	0	4 (22)	4 (66)	
Missing	15	0	13	1	1	
MFI (years)						0.37
0	11 (15)	3 (43)	4 (10)	3 (16)	1 (14.5)	
>0-3	8 (11)	0	4 (10)	3 (16)	1 (14.5)	
>3	55 (74)	4 (57)	33 (80)	13 (68)	5 (71)	
Number of metastatic sites						0.92
1–2	56 (76)	5 (71)	30 (73)	15 (79)	6 (86)	
≥3	18 (24)	2 (29)	11 (27)	4 (21)	1 (14)	
Metastatic site						0.18
Non-visceral	39 (53)	5 (71)	20 (49)	8 (42)	6 (86)	
Visceral	35 (47)	2 (29)	21 (51)	11 (58)	1 (14)	
ER (DM)						<0.001
Negative	12 (16)	0 (0)	0 (0)	7 (37)	5 (71)	
Positive	61 (84)	6 (100)	41 (100)	12 (63)	2 (29)	
Missing	1	1	0	0	0	
PR (DM)						0.017
Negative	42 (58)	4 (67)	17 (43)	15 (79)	6 (86)	
Positive	30 (42)	2 (33)	23 (57)	4 (21)	1 (14)	
Missing	2	1	1	0	0	
HER2 (DM)						0.006
Negative	61 (88)	5 (100)	39 (97)	12 (67)	5 (83)	
Positive	8 (12)	0 (0)	1 (3)	6 (33)	1 (17)	
Missing	5	2	1	1	1	
Number of CTCs (BL)						0.24
<5	29 (39)	4 (57)	12 (29)	9 (47)	4 (57)	
≥5	45 (61)	3 (43)	29 (71)	10 (53)	3 (43)	

Missing	0

Abbreviations: BL, baseline; CTCs, circulating tumor cell; DM, distant metastasis; ECOG, Eastern Cooperative Oncology Group; ER, estrogen receptor; HER2, human epidermal growth factor receptor 2; HER2-E, human epidermal growth factor receptor 2 enriched; MBC, metastatic breast cancer; MFI, metastasis-free interval; NHG, Nottingham Histological Grade; *n*, number; PT, primary tumor PR, progesterone receptor. [a]*P*-value from Fisher's exact test; [b]At primary diagnosis.

Table S4. Changes in PAM50 breast cancer intrinsic subtype between PT, LNM, and DM.

PT	LNM	DM	*n*
Lum A	Lum A	Lum A	2
Lum A	Lum A	Lum B	5
Lum A	Lum A	HER2-E	0
Lum A	Lum A	Basal-like	0
Lum A	Lum B	Lum A	1
Lum A	Lum B	Lum B	6
Lum A	Lum B	HER2-E	4
Lum A	Lum B	Basal-like	2
Lum A	HER2-E	Lum A	0
Lum A	HER2-E	Lum B	0
Lum A	HER2-E	HER2-E	0
Lum A	HER2-E	Basal-like	0
Lum A	Basal-like	Lum A	0
Lum A	Basal-like	Lum B	0
Lum A	Basal-like	HER2-E	0
Lum A	Basal-like	Basal-like	0
Lum B	Lum A	Lum A	0
Lum B	Lum A	Lum B	0
Lum B	Lum A	HER2-E	0
Lum B	Lum A	Basal-like	0
Lum B	Lum B	Lum A	0
Lum B	Lum B	Lum B	7
Lum B	Lum B	HER2-E	1
Lum B	Lum B	Basal-like	0
Lum B	HER2-E	Lum A	0
Lum B	HER2-E	Lum B	0
Lum B	HER2-E	HER2-E	0
Lum B	HER2-E	Basal-like	0
Lum B	Basal-like	Lum A	0
Lum B	Basal-like	Lum B	0
Lum B	Basal-like	HER2-E	1
Lum B	Basal-like	Basal-like	0
HER2-E	Lum A	Lum A	0
HER2-E	Lum A	Lum B	0
HER2-E	Lum A	HER2-E	0
HER2-E	Lum A	Basal-like	0
HER2-E	Lum B	Lum A	0
HER2-E	Lum B	Lum B	0
HER2-E	Lum B	HER2-E	0
HER2-E	Lum B	Basal-like	0
HER2-E	HER2-E	Lum A	0
HER2-E	HER2-E	Lum B	0
HER2-E	HER2-E	HER2-E	2
HER2-E	HER2-E	Basal-like	0
HER2-E	Basal-like	Lum A	0
HER2-E	Basal-like	Lum B	0

HER2-E	Basal-like	HER2-E	0
HER2-E	Basal-like	Basal-like	0
Basal-like	Lum A	Lum A	0
Basal-like	Lum A	Lum B	0
Basal-like	Lum A	HER2-E	0
Basal-like	Lum A	Basal-like	0
Basal-like	Lum B	Lum A	0
Basal-like	Lum B	Lum B	0
Basal-like	Lum B	HER2-	0
Basal-like	Lum B	Basal-like	1
Basal-like	HER2-E	Lum A	0
Basal-like	HER2-E	Lum B	0
Basal-like	HER2-E	HER2-E	0
Basal-like	HER2-E	Basal-like	0
Basal-like	Basal-like	Lum A	0
Basal-like	Basal-like	Lum B	0
Basal-like	Basal-like	HER2-E	0
Basal-like	Basal-like	Basal-like	1

Abbreviations: DM, distant metastasis; HER2-E, human epidermal growth factor receptor 2 enriched; LNM, lymph node metastasis; n, number; PT, primary tumor

Table S5. Overall PAM50 breast cancer intrinsic subtype shifts from primary tumors to distant metastases in relation to adjuvant therapy given.

**Adjuvant Therapy**		***n***	**Subtype Stable *n* (%)**	**Subtype Shift *n* (%)**	***p***
Chemotherapy	Yes	27	12(44)	15 (56)	0.61
	No	34	18 (53)	16 (47)	
Endocrine therapy	Yes	44	17 (39)	27 (61)	0.01
	No	17	13 (76)	4 (24)	
Endocrine therapy (in PT luminal subtype only)	Yes	40	29 (73)	11 (27)	0.25
	No	12	11 (92)	1 (8)	

Abbreviations: *n*, number; PT, primary tumor
